# Selection and Evaluation of Potential Reference Genes for Gene Expression Analysis in the Brown Planthopper, *Nilaparvata lugens* (Hemiptera: Delphacidae) Using Reverse-Transcription Quantitative PCR

**DOI:** 10.1371/journal.pone.0086503

**Published:** 2014-01-23

**Authors:** Miao Yuan, Yanhui Lu, Xun Zhu, Hu Wan, Muhammad Shakeel, Sha Zhan, Byung-Rae Jin, Jianhong Li

**Affiliations:** 1 Laboratory of Pesticide, College of Plant Science & Technology, Huazhong Agricultural University, Wuhan, China; 2 Laboratory of Insect Molecular Biology and Biotechnology, Department of Applied Biology, College of Natural Resources and Life Science, Dong-A University, Busan, Korea; Zhejiang University, China

## Abstract

The brown planthopper (BPH), *Nilaparvata lugens* (Hemiptera, Delphacidae), is one of the most important rice pests. Abundant genetic studies on BPH have been conducted using reverse-transcription quantitative real-time PCR (qRT-PCR). Using qRT-PCR, the expression levels of target genes are calculated on the basis of endogenous controls. These genes need to be appropriately selected by experimentally assessing whether they are stably expressed under different conditions. However, such studies on potential reference genes in *N. lugens* are lacking. In this paper, we presented a systematic exploration of eight candidate reference genes in *N. lugens*, namely, actin 1 (ACT), muscle actin (MACT), ribosomal protein S11 (RPS11), ribosomal protein S15e (RPS15), alpha 2-tubulin (TUB), elongation factor 1 delta (EF), 18S ribosomal RNA (18S), and arginine kinase (AK) and used four alternative methods (BestKeeper, geNorm, NormFinder, and the delta Ct method) to evaluate the suitability of these genes as endogenous controls. We examined their expression levels among different experimental factors (developmental stage, body part, geographic population, temperature variation, pesticide exposure, diet change, and starvation) following the MIQE (Minimum Information for publication of Quantitative real time PCR Experiments) guidelines. Based on the results of RefFinder, which integrates four currently available major software programs to compare and rank the tested candidate reference genes, RPS15, RPS11, and TUB were found to be the most suitable reference genes in different developmental stages, body parts, and geographic populations, respectively. RPS15 was the most suitable gene under different temperature and diet conditions, while RPS11 was the most suitable gene under different pesticide exposure and starvation conditions. This work sheds light on establishing a standardized qRT-PCR procedure in *N. lugens*, and serves as a starting point for screening for reference genes for expression studies of related insects.

## Introduction

The brown planthopper (BPH), *Nilaparvata lugens* (*N. lugens*), is the most devastating rice pest in extensive areas throughout Asia [1]. The BPH ingests nutrients specifically from the phloem of rice plants with its stylet, causing the entire plant to become yellow and dry rapidly, a phenomenon referred to as hopperburn [2]. In addition, BPH is a vector of viruses that cause diseases in rice, such as *Rice ragged stunt virus* (RRSV) and *Rice grassy stunt virus* (RGSV) [3]. In recent years, *N. lugens* outbreaks have occurred more frequently in the Yangtze River Delta areas and in the South of China [4], [5]. Because of its long-distance migration, quick adaptation to resistant rice varieties and development of high resistance to pesticides, *N. lugens* infestations are difficult to control [6].

Quantitative real-time reverse-transcription polymerase chain reaction (qRT-PCR) is the most sensitive and accurate method to measure variations in mRNA expression levels of a single gene in different experimental and clinical conditions [7], [8]. At present, RNA interference (RNAi) is an effective tool to control important insect pests via gene silencing [9], [10], [11], [12], [13]. Interestingly, several studies have shown that injection or ingestion of dsRNAs in *N. lugens* can reduce the transcript levels of target genes [14], [15], [16]. On the other hand, the sequencing of *N.lugens* genome has been recently included in the 5000 insect genome initiative (http://arthropodgenomes.org/wiki/i5K), somehow reflecting the economic importance of this pest. Meanwhile, enormous progress has been made by means of the sequencing of *N. lugens* ESTs from various tissues [17], transcriptome analysis [18], and pyrosequencing the midgut transcriptome [19]. These data provided comprehensive gene expression information at the transcriptional level that could facilitate our understanding of the molecular mechanisms underlying various physiological aspects including development, wing dimorphism and sex difference in BPH. For precise and reliable gene expression results, normalization of quantitative real-time PCR data is required against a control gene, which is typically a gene that shows highly uniform expression in living organisms during various phases of development under different environmental or experimental conditions [20]. Quantitative assays frequently use housekeeping genes such as β-actin, glyceraldehyde-3-phosphate dehydrogenase (GAPDH), tubulin, and 18S ribosomal RNA (rRNA) because they are necessary for survival and are synthesized in all nucleated cell types. It is often considered that there are only a few fluctuations in the transcription of these genes compared to others [21], [22], [23]. However, numerous studies show that the expression levels of these housekeeping genes also vary in different situations [24], [25].

Although qRT-PCR is a highly reliable method for measuring gene transcript levels, if the reference genes are not selected properly, it will result in inaccurate calculation of the normalization factor and consequently obscure actual biological differences among samples. Therefore, it is necessary to validate the expression stability of control genes under specific experimental conditions before using them for normalization. Reference genes in qRT-PCR studies on BPH have often been selected based on consensus and experience in other species rather than empirical evidence in support of their efficacy [1], [14], [15], [16]. There is therefore a definite need to analyze the expression of these genes in different body parts in different populations, under different experimental conditions, and at different stages of development. This study examined the stability of eight reference genes, actin 1 (ACT), muscle actin (MACT), ribosomal protein S11 (RPS11), ribosomal protein S15e (RPS15), alpha 2-tubulin (TUB), elongation factor 1 delta (EF), 18S ribosomal RNA (18S), and arginine kinase (AK), in *N. lugens* in terms of different factors (developmental stage, body part, geographic population, temperature variation, pesticide treatment, diet change, and starvation).

## Materials and Methods

### Insects

Unless stated, the laboratory population of *N. lugens* was originally collected from Changsha, Hunan, People’s Republic of China in 2009 and artificially maintained in our lab since. The laboratory strain and other populations used in this experiment are from different fields which no specific permissions were required, because these fields are the experimental plots of Huazhong Agricultural University, Wuhan, Hubei, China. The insects were reared on rice (Shanyou 63) in a thermostatic chamber. The chamber was maintained at 80% relative humidity, 25°C±2°C temperature and a 14∶10 h light:dark cycle.

### Treatments

1. Developmental stage: For each treatment group, 6 samples each of about 50 one-day-old eggs, 50 1^st^ instar nymphs, 30 2^nd^ instar nymphs, 20 3^rd^ instar nymphs, 20 4^th^ instar nymphs, 20 5^th^ instar nymphs, 20 adult females, and 20 adult males of *N. lugens* were collected.
2. Body part: A dissection needle and a tweezer (Dumont, World Precision Instruments, USA) were used to obtain head, thorax, and abdomen from virgin adult males and females from the *N. lugens* laboratory population. Besides, virgin adult males and females were collected as whole-body samples. For each treatment group, 6 samples of 20 insects each were collected.
3. Geographic population: One geographic population was originally collected from Changsha, Hunan, China, which was maintained with no exposure to insecticides. The other population was generously provided by Dr. Manqun Wang (Huazhong Agricultural University), which was originally collected from Wuhan, Hubei, China. These two places are approximately 310 kilometers apart. Both these populations have been maintained for more than 3 years in our laboratory. Third instar nymphs and adults were collected. For each treatment group, 6 samples of 20 insects each were collected.
4. Temperature-induced stress: Third instar nymphs were divided into 10 groups and then each group was exposed for 5 min to each temperature: extremely low temperatures (4°C, 8°C, and 12°C), low temperatures (16°C and 20°C), average temperatures (24°C and 28°C), and high temperatures (32°C, 36°C and 40°C). For each treatment group, 6 samples of 20 insects each were collected. There was no mortality in response to the temperature treatment.
5. Pesticide-induced stress: The stability of candidate reference genes was tested in 3^rd^ instar nymphs subjected to 6 different pesticide treatments: compound pesticide (abamectin 3.6 mg/L+nitenpyram 0.2 mg/L), nitenpyram (0.4 mg/L), pymetrozine (42.08 mg/L), buprofezin (1.19 mg/L), isoprocarb (34.91 mg/L), and chlorpyrifos (52.27 mg/L). The concentration of pesticide was LC_50_ and opted by the results of bioassay (Table S1). The testing pesticide solutions were made using water containing 0.1% w/v Triton X-100 (Beijing Solarbio Science and Technology Co. Ltd., China). The roots of the rice seedlings were tightly packaged by the absorbent cotton. The seedlings were completely dipped in the testing solutions for 5 s and then air dried for 10–15 min depending on the ambient relative humidity (http://www.irac-online.org/content/uploads/2009/09/Method_005_v3_june09.pdf). Third instar nymphs were collected from the laboratory population and then transferred into the transparent plastic tube which contained the testing seedlings. Water containing 0.1% w/v Triton X-100 was used as a separate control group for each pesticide treatment. Because of the different mechanism of action of the testing pesticide, the living insects were collected after 4, 4, 7, 5, 3 and 3 days for compound pesticide, nitenpyram, pymetrozine, buprofezin, isoprocarb, and chlorpyrifos treatments, respectively [26], [27], [28]. For each treatment group, 6 samples of 50 insects each were collected.
6. Diet-induced stress: Our third treatment condition involved the stability of reference gene expression in *N. lugens* challenged with different diets: artificial diet [29], Taichung Native 1 rice (TN1), Minghui 63 rice (MH63), transgenic rice Huahui 1 rice (HH1), Shanyou 63 rice (SY63), and transgenic rice Bt Shanyou 63 rice (BTSY63). The seeds of TN1, MH63, HH1, SY63, and BTSY63 were generously provided by Dr. Yongjun Lin (Huazhong Agricultural University). Newly hatched nymphs were collected and then reared on different diets. From each diet group, 3^rd^ instar nymphs and adults were collected. For each treatment group, 6 replications of 20 insects each were collected.
7. Starvation-induced stress: Third instar nymphs and adults were collected in separate glass cylinders (15.0 cm in length and 2.5 cm in diameter) covered by Parafilm M (Bemis, USA) with no food in a thermostatic chamber; they were kept there for two days. We used a satiation group (3^rd^ instar nymphs and adults fed on SY63) as the control group. For each treatment group, 6 samples of 50 insects each were collected. The mortality rate was approximately 30%.

### Total RNA Extraction and cDNA Synthesis

All collected insects were preserved in a clean micro-centrifuge tube (1.5 ml) and stored at −80°C after freezing in liquid nitrogen. Six total RNA samples were prepared for each developmental and treatment group. Subsequently, total RNA was extracted using a SV Total RNA Isolation System (Promega, USA). According to the manufacturer’s protocol, total RNA was incubated for 15 min at 20–25°C after adding 5 µl DNase I enzyme (Promega, USA). The quality and quantity of RNA were assessed with a UV-1800 spectrophotometer (SHIMADZU, Japan). Only samples with a 260/280 ratio of 1.9 to 2.1, which indicates no protein contamination, and a 260/230 ratio of 2.0 to 2.4, which indicates no guanidine thiocyanate contamination were considered. Total RNA concentration ranged from 447 to 1071 ng/µl according to spectrophotometric determination. The A_260_:A_280_ values of the isolated total RNA ranged from 1.914 to 1.966, indicating the high purity of the total RNA. The integrity of total RNA was confirmed by 1% agarose gel electrophoresis. CDNA was produced using the PrimeScript 1^st^ Strand cDNA Synthesis Kit (TAKARA, Japan) in a total volume of 20 µl, with 4 µl 5×PrimeScript Buffer,1 µg of total RNA, 1 µl oligo dT primer, 1 µl PrimeScript RTase (200 U/µl), and 0.5 µl RNase Inhibitor (40 U/µl). Following the manufacturer’s protocol, the 20 ul mixture was incubated for 60 min at 42°C. No-template and no-reverse-transcription controls were included for each reverse-transcription run for the control treatment. CDNA was stored at −20°C for later use.

### Primer Design

The sequences of all candidate reference genes were downloaded from GenBank (http://www.ncbi.nlm.nih.gov/genbank/) and UNKA (BPH) EST BLAST database (http://bphest.dna.affrc.go.jp/). The PCR primer sequences used for quantification of the expression of the genes encoding ACT, MACT, RPS11, RPS15, TUB, EF, 18S, and AK are shown in Table 1. The secondary structure of the template was analyzed with UNAFold using the DNA folding form of the mfold web server (http://mfold.rna.albany.edu/?q=mfold/DNA-Folding-Form) [30] with the following settings: melting temperature, 60°C; DNA sequence, linear; Na^+^ concentration, 50 mM; Mg^2+^ concentration, 3 mM. The other parameters were set by default. The primers were designed on the NCBI-Primer-BLAST website (http://www.ncbi.nlm.nih.gov/tools/primer-blast/index.cgi?LINK_LOC=BlastHome). The settings in NCBI-Primer-BLAST were as follows: primer melting temperature, 57–63°C; primer GC content, 40–60%; and PCR product size, 150–300 base pairs. The excluded regions were determined using mfold, and the other parameters were set by default. Four primer pairs were designed for each gene. The length of PCR products was assessed using gel electrophoresis, and the identity of the PCR products was confirmed by sequence analysis. Only primers which could not amplify non-specific products and dimmers were employed. A 10-fold dilution series of cDNA from the whole body of adults was employed as a standard curve, and the reverse-transcription qPCR efficiency was determined for each gene and each treatment, using the linear regression model [31]. The corresponding qRT-PCR efficiencies (E) were calculated according to the equation: E = (10^[−1/slope]^−1)×100 [32]. After detecting the efficiencies of the chosen primers, the primers which displayed a coefficient of correlation greater than 0.99 and efficiencies between 95% and 108% were selected for the next qRT-PCR (Table 1).

**Table 1:** Function, primer sequence and amplicon characteristics of the candidate reference genes used in this study.

Gene symbol	Gene name	(putative) Function	Gene ID	Primer sequences [5′→3′]	L (bp)^a^	E (%)^b^	R^2c^
ACT	actin 1	Involved in cell motility,	ABY48093.1	*For 5′* TGCGTGACATCAAGGAGAAG *3′*	283	96.7	0.997
		structure and integrity		*Rev 5′* GTACCACCGGACAGGACAGT *3′*			
MACT	muscle actin	Involved in cell motility,	ADB92676.1	*For 5′* CTTGGCTGGTCGTGACTTGACCGA *3′*	179	101.7	0.997
		structure and integrity		*Rev 5′* ACTTCTCCAGGGAGGTGGAGGCG *3′*			
RPS11	ribosomal protein S11	Structural constituent of	ACN79505.1	*For 5′* CCGATCGTGTGGCGTTGAAGGG *3′*	159	93.5	0.997
		ribosome		*Rev 5′* ATGGCCGACATTCTTCCAGGTCC *3′*			
RPS15	ribosomal protein S15	Structural constituent of	ACN79501.1	*For 5′* TAAAAATGGCAGACGAAGAGCCCAA *3′*	150	101.5	0.999
		ribosome		*Rev 5′* TTCCACGGTTGAAACGTCTGCG *3′*			
TUB	α-tubulin	Cytoskeleton structural	ACN79512.1	*For 5′* ACTCGTTCGGAGGAGGCACC *3′*	174	101.7	0.995
		protein		*Rev 5′* GTTCCAGGGTGGTGTGGGTGGT *3′*			
EF	elongation factor 1 delta	Structural constituent of	DQ445523.1	*For 5′* GAAGTAGCTCTGGCACAGGA *3′*	150	103.9	0.996
		ribosome		*Rev 5′* TTGACGAGCCTTTGCTACCT *3′*			
18S	18S ribosomal RNA	Cytosolic small ribosomal	JN662398.1	*For 5′* GTAACCCGCTGAACCTCC *3′*	170	107.2	0.990
		subunit		*Rev 5′* GTCCGAAGACCTCACTAAATCA *3′*			
AK	arginine kinase	Key enzyme for cellular	AAT77152.1	*For 5′* ACCACAACGACAACAAGACCTTCC *3′*	186	98.3	0.998
		energy metabolism		*Rev 5′* TGGGACAGAAAGTCAGGAATCCCA *3′*			

a Length of the amplicon.

b Real-time qPCR efficiency (calculated by the standard curve method).

c Reproducibility of the real-time qPCR reaction.

### Reverse-transcription qPCR Assays

Triplicate 1^st^-strand DNA aliquots for each treatment served as templates for qRT-PCR using SsoFast™ EvaGreen® Supermix (Bio-Rad) on a Bio-Rad iQ2 Optical System (Bio-Rad). Amplification reactions were performed in a 20 µl volume with 1 µl of cDNA and 100 nM of each primer, in iQ™ 96-well PCR plates (Bio-Rad) covered with Microseal “B” adhesive seals (Bio-Rad). Thermal cycling conditions were as follows: initial denaturation temperature, 95°C for 30 s, followed by 40 cycles at 95°C for 5 s and 60°C for 10 s. After the reaction, a melting curve analysis from 65°C to 95°C was applied to ensure consistency and specificity of the amplified product.

### Data Mining and Selection of Reference Genes

Expression levels were determined as the number of cycles needed for the amplification to reach a fixed threshold in the exponential phase of the PCR reaction [33]. The number of cycles is referred to as the threshold cycle (Ct) value. The threshold was set at 500 for all genes. Four freely available software tools, BestKeeper [34], geNorm version3.5 [35], NormFinder version 0.953 [36], and the delta Ct method [37] were used to evaluate gene expression stability. The Excel based tool Bestkeeper, uses raw data (Ct values) and PCR efficiency (E) to determine the best-suited standards and combines them into an index by the coefficient of determination and the P value [34]. Quantities transformed to a linear scale (the highest relative quantity for each gene was set to 1) were used as input data for geNorm and NormFinder. geNorm algorithm first calculates an expression stability value (M) for each gene and then compares the pairwise variation (V) of this gene with the others. Reference genes are ranked according to their expression stability by a repeated process of stepwise exclusion of the least stably expressed genes. The geNorm program also indicates the minimum number of reference genes for accurate normalization by the pairwise variation value. The value of Vn/n+1 under 0.15 means that no additional genes are required for normalization [35]. NormFinder provides a stability value for each gene which is a direct measure for the estimated expression variation enabling the user to evaluate the systematic error introduced when using the gene for normalization [36]. The delta Ct method compares relative expression of pairs of genes within each sample to confidently identify useful housekeeping genes [37]. A user-friendly web-based comprehensive tool, RefFinder (http://www.leonxie.com/referencegene.php?type=reference) was used, integrating four currently available major software programs to compare and ranking the tested candidate reference genes. Based on the rankings from each program, RefFinder assigns an appropriate weight to an individual gene and calculates the geometric mean of their weights for the overall final ranking. According to the results of RefFinder, candidate genes with the lower ranking were considered to be most stably expressed under tested experimental conditions, and thus could be selected as ideal reference genes.

## Results

### Expression Profiles of Candidate Reference Genes

**Figure 1:**
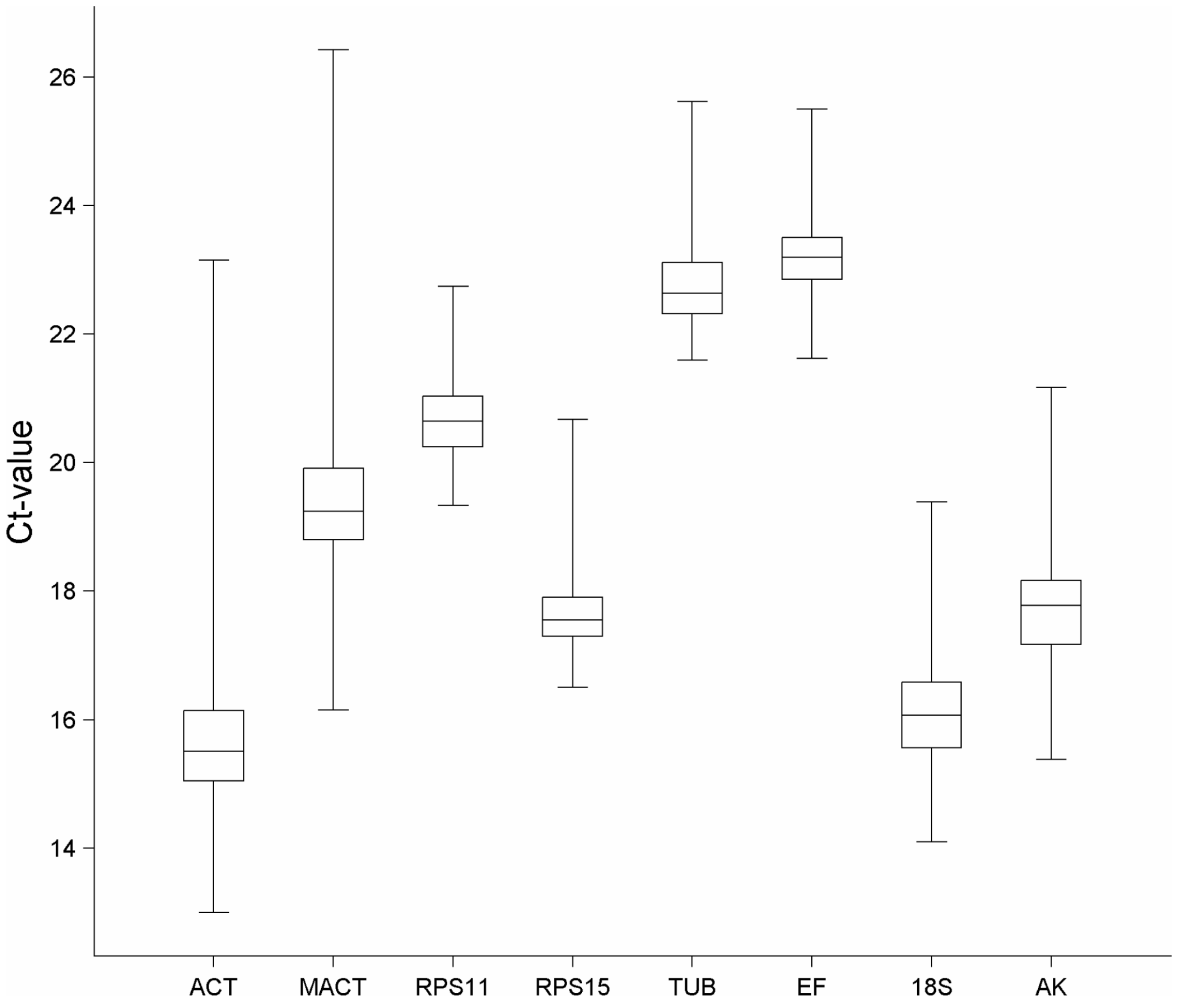
Expression levels of candidate reference genes. The expression level of candidate *N. lugens* reference genes in the total samples is shown in terms of the cycle threshold number (Ct-value). The data are expressed as whisker box plots; the box represents the 25^th^–75^th^ percentiles, the median is indicated by a bar across the box, the whiskers on each box represent the minimum and maximum values.

In order to evaluate gene expression levels of all studied housekeeping genes within the whole sample set of *N.lugens*, mRNA expressions for every gene were measured. Gene expression levels showed a broad range of variance between Ct-value 12.99 (ACT) and 26.43 (MACT) (Figure 1). Out of eight studied genes, ACT (mean Ct-value 15.71) and 18S (mean Ct-value 16.16) were expressed at the highest levels; TUB (mean Ct-value 22.79) and EF (mean Ct-value 23.25) at the lowest levels. The lowest expression variability within all samples was observed for the gene RPS11 (mean Ct-value±SD, 20.65±0.58) and RPS15 (17.74±0.69). ACT (15.71±1.36) and MACT (19.37±1.39) showed the most variable expression within the sample set.

### Analysis of Gene Expression Stability

**Figure 2:**
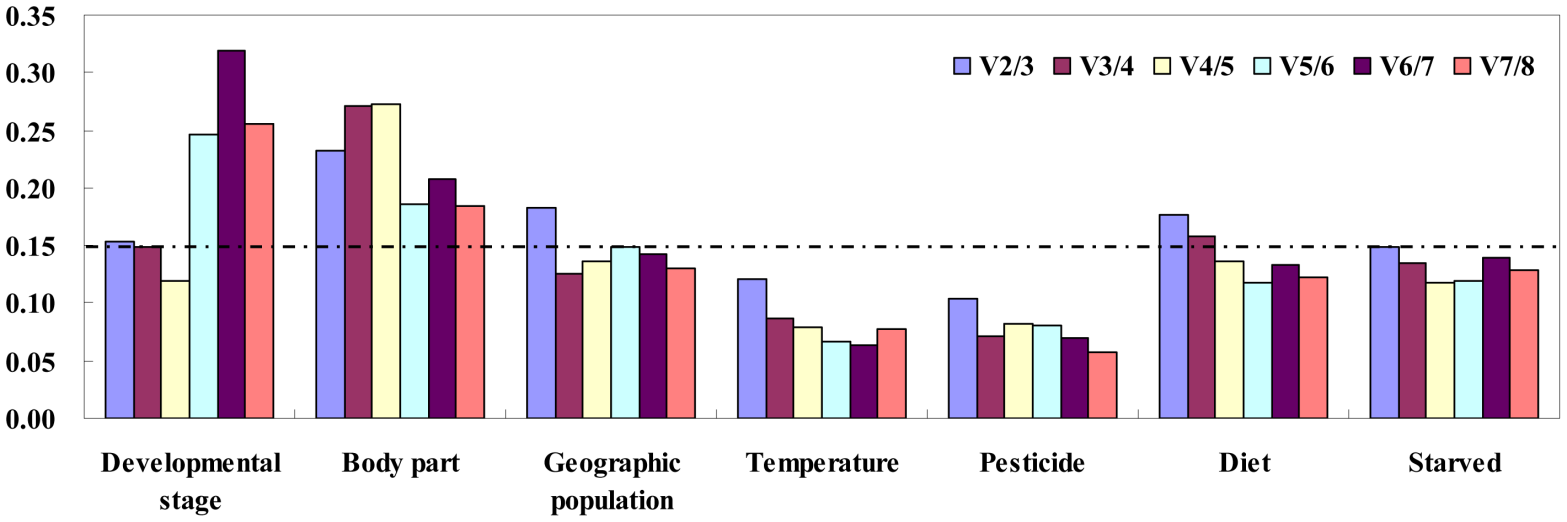
Determination of the optimal number of reference genes for accurate normalization calculated by geNorm. The value of Vn/Vn+1 indicates the pairwise variation (Y axis) between two sequential normalization factors and determines the optimal number of reference genes required for accurate normalization. A value below 0.15 indicates that an additional reference gene will not significantly improve normalization.

1. Developmental stage: The stability ranking generated by the Delta Ct method was largely similar with the results obtained from BestKeeper and NormFinder. However, the most stable genes ranking by geNorm analysis were different to the results generated by the other three methods. All four programs identified ACT and MACT as the least stable genes, and RPS11, RPS15, and EF as the most stable genes except geNorm (Table 2). According to the results of RefFinder, the stability ranking from the most stable to the least stable in the developmental stages was RPS15, RPS11, TUB, EF, 18S, AK, ACT, and MACT (Table S2). As can be noticed, TUB was the most stable gene across different nymphal stages and across different sexes (Table S3). With geNorm, the V value of 0.154 obtained for the RPS15-RPS11 pair was near the proposed cut-off value of 0.15. Moreover, the inclusion of additional reference genes did not lower the V value below the proposed 0.15 cut-off value until the fourth gene was added (Figure 2). According to geNorm, four reference genes (RPS15, TUB, 18S, and EF) should be required for a suitable normalization in the different developmental stages.
2. Body part: All four programs, except BestKeeper, identified RPS11, RPS15, and 18S as the most stable genes (Table 2). According to the results of RefFinder, the stability ranking from the most stable to the least stable gene in different body parts was RPS11, TUB, RPS15, 18S, ACT, MACT, EF, and AK (Table S2). RPS11 was the most stable gene across the different body parts of female and male adults (Table S4). TUB was the most stable gene between males and females in the head, thorax, and whole body (Table S5). However, TUB displayed high instability between males and females in the abdomen (Table S5). GeNorm analysis revealed that the pairwise variation values were all above the cut-off value and decreased with the added reference genes (Figure 2). These results indicated that normalization with three stable reference genes (RPS11, 18S, and RPS15) was required (as suggested by the geNorm manual).
3. Population: The stability ranking generated by the Delta Ct method was largely similar with the results obtained by NormFinder. All four programs, except geNorm, identified TUB as the most stable gene (Table 2). According to the results of RefFinder, the stability ranking from the most stable to the least stable gene in the two different populations was TUB, RPS11, EF, RPS15, AK, ACT, 18S, and MACT (Table S2). EF and TUB showed high expression stability in the nymphs and adults of these two populations, respectively. Interestingly, RPS15 showed high instability in the adults of both different populations, and was ranked one of the least stable genes in the 3^rd^ instar nymphs of two different populations (Table S6). GeNorm analysis revealed that all the pairwise variation values were below the proposed 0.15 cut-off, except for V2/3 (Figure 2). According to geNorm, three reference genes (RPS11, EF, and RPS15) should be required for a suitable normalization in these two different geographic populations.
4. Temperature: All four programs identified RPS15 and TUB as the most stable genes, and identified ACT as the least stable gene (Table 2). From the results of RefFinder, the stability ranking from the most stable to the least stable gene in the temperature-stressed samples was RPS15, TUB, EF, RPS11, AK, MACT, 18S, and ACT (Table S2). Under extremely low temperature stress, AK was ranked one of the most stable genes, while it was ranked one of the least stable genes under low temperature stress (Table S7). TUB was the most stable gene at average temperatures (Table S7). MACT, which was ranked one of the least stable genes under extremely low temperature, low temperature, and average temperature, showed high expression stability under high-temperature stress (Table S7). ACT was ranked as the least stable gene in all temperature conditions (Table S7). GeNorm analysis revealed that all the pairwise variation values were below the proposed 0.15 cut-off (Figure 2). According to geNorm, three reference genes (RPS15, TUB, and EF) should be required for a suitable normalization in the different temperature treatment samples.
5. Pesticide treatment: The stability ranking generated by the Delta Ct method was same as the results obtained from NormFinder and geNorm. The stability ranking generated by BestKeeper was largely similar with the one obtained by the other three methods. All four programs identified RPS11 and EF as the most stable genes (Table 2). According to RefFinder, the stability ranking from the most stable to the least stable in the pesticide-stressed samples was RPS11, EF, TUB, RPS15, 18S, AK, MACT, and ACT (Table S2). As can be noticed, RPS11 was also the most stable gene in all pesticide-treated samples (Table S2), compound-pesticide-treated samples, buprofezin-treated samples, and isoprocarb-treated samples (Table S8). EF and TUB were the most stable genes in the nitenpyram-treated samples and chlorpyrifos-treated samples (Table S8), respectively. MACT, which was ranked one of the least stable genes in other pesticide treatments, showed the highest stability in pymetrozine-treated samples (Table S8). GeNorm analysis revealed that all the pairwise variation values were below the proposed 0.15 cut-off value (Figure 2). According to geNorm, three reference genes (RPS11, EF, and TUB) should be required for a suitable normalization in the pesticide-stressed samples.
6. Diet: All four programs identified RPS15 as the most stable gene, and identified ACT and MACT as the least stable genes (Table 2). According to RefFinder, the stability ranking from the most stable to the least stable in the different diets treatments was RPS15, TUB, RPS11, EF, AK, 18S, ACT, and MACT (Table S2). RPS15 was the most stable gene in *N. lugens* reared on artificial diet, TN1, HH1 and SY63, and was ranked second in the *N. lugens* reared on MH63 (Table S9). However, RPS15 was the least stable gene in *N. lugens* reared on BTSY63 (Table S9). The results also showed that RPS15 and RPS11 were the most stable genes in *N. lugens* reared on non-genetically modified rice and genetically modified rice, respectively (Table S10). In *N. lugens* nymphs reared on non-genetically modified rice, TUB was the most stable gene (Table S10), while in *N. lugens* adults reared on non-genetically modified rice, RPS15 was still the most stable gene (Table S10). RPS15 and 18s were the most stable genes in the *N. lugens* nymphs and adults reared on genetically modified rice, respectively (Table S10). With geNorm, the V value of 0.176 obtained by the RPS15 and TUB pair was near the proposed 0.15 cut-off value. Moreover, the inclusion of additional reference genes did not lower the V value below the proposed 0.15 cut-off until the 4^th^ gene was added (Figure 2). According to geNorm, four reference genes (RPS15, TUB, EF and RPS11) should be required for a suitable normalization in the different diets treatments.
7. Starvation: The gene stability of the starvation group compared to a satiation group (SY63) was analyzed. All four programs identified ACT and MACT as the least stable genes, and identified RPS11 as the most stable gene except BestKeeper (Table 2). According to RefFinder, the stability ranking from the most stable to the least stable in the starvation treatments was RPS11, TUB, RPS15, AK, 18S, EF, ACT, and MACT (Table S2). RPS11 was the most stable gene both in starved nymphs and starved adults (Table S11). GeNorm analysis revealed that all the pairwise variation values were below the proposed 0.15 cut-off (Figure 2). According to geNorm, three reference genes (RPS11, AK, and EF) should be required for a suitable normalization in the starvation treatments.

**Table 2:** Ranking order of the candidate reference genes of *N. lugens* in different experimental conditions.

		Delta Ct		BestKeeper		NormFinder		geNorm	
Experimentalconditions	Rank	Genename	Standarddeviation	Genename	Standarddeviation	Genename	Stabilityvalue	Genename	Stabilityvalue
Different	1	RPS11	1.190	RPS11	0.380	RPS11	0.407	RPS15/TUB	0.425
developmental	2	RPS15	1.204	RPS15	0.520	RPS15	0.705		
stages	3	EF	1.274	EF	0.541	EF	0.827	18S	0.480
	4	TUB	1.355	18S	0.557	AK	0.876	EF	0.566
	5	18S	1.401	TUB	0.605	TUB	1.069	RPS11	0.614
	6	AK	1.532	AK	0.816	18S	1.144	AK	0.915
	7	ACT	2.047	MACT	1.539	ACT	1.864	ACT	1.309
	8	MACT	2.148	ACT	1.582	MACT	2.004	MACT	1.519
Different body parts	1	RPS11	1.096	RPS15	0.465	RPS11	0.203	RPS11/18S	0.620
	2	RPS15	1.210	TUB	0.501	18S	0.628		
	3	18S	1.212	RPS11	0.557	RPS15	0.741	RPS15	0.717
	4	ACT	1.427	AK	0.928	TUB	1.093	TUB	0.935
	5	TUB	1.455	EF	0.953	ACT	1.100	EF	1.149
	6	MACT	1.458	18S	0.963	MACT	1.152	ACT	1.193
	7	EF	1.610	ACT	1.001	AK	1.411	MACT	1.294
	8	AK	1.703	MACT	1.013	EF	1.421	AK	1.396
Different geographic	1	TUB	0.708	TUB	0.590	TUB	0.145	RPS11/EF	0.212
populations	2	RPS11	0.728	EF	0.637	RPS11	0.362		
	3	RPS15	0.774	RPS15	0.637	RPS15	0.412	RPS15	0.440
	4	EF	0.785	RPS11	0.706	EF	0.506	TUB	0.501
	5	AK	0.922	ACT	0.756	AK	0.709	AK	0.594
	6	ACT	0.936	AK	0.794	ACT	0.750	ACT	0.707
	7	MACT	1.122	MACT	0.824	18S	1.016	MACT	0.803
	8	18S	1.156	18S	0.980	MACT	1.017	18S	0.891
Temperature-stress	1	RPS15	0.433	RPS15	0.204	RPS15	0.221	RPS15/TUB	0.287
treatments	2	TUB	0.450	TUB	0.235	TUB	0.265		
	3	EF	0.478	RPS11	0.277	EF	0.305	EF	0.356
	4	RPS11	0.500	AK	0.282	MACT	0.342	AK	0.379
	5	AK	0.501	MACT	0.325	AK	0.345	RPS11	0.408
	6	MACT	0.505	18S	0.345	RPS11	0.351	MACT	0.429
	7	18S	0.544	ACT	0.357	18S	0.414	18S	0.454
	8	ACT	0.688	EF	0.547	ACT	0.608	ACT	0.512
Pesticide-stress	1	RPS11	0.435	EF	0.245	RPS11	0.253	RPS11/EF	0.277
treatments	2	EF	0.435	RPS11	0.248	EF	0.257		
	3	TUB	0.439	TUB	0.267	TUB	0.271	TUB	0.318
	4	RPS15	0.445	RPS11	0.296	RPS15	0.277	RPS15	0.328
	5	18S	0.518	MACT	0.465	18S	0.391	18S	0.379
	6	AK	0.544	AK	0.473	AK	0.430	AK	0.430
	7	MACT	0.557	ACT	0.539	MACT	0.443	MACT	0.469
	8	ACT	0.557	18S	0.583	ACT	0.443	ACT	0.491
Different diet	1	RPS15	0.730	RPS15	0.490	RPS15	0.362	RPS15/TUB	0.421
treatments	2	TUB	0.792	RPS11	0.527	TUB	0.485		
	3	RPS11	0.850	EF	0.565	RPS11	0.559	EF	0.513
	4	EF	0.851	AK	0.584	AK	0.578	RPS11	0.603
	5	AK	0.872	TUB	0.603	EF	0.626	18S	0.670
	6	18S	0.906	18S	0.639	18S	0.666	AK	0.723
	7	ACT	0.989	ACT	0.658	ACT	0.778	ACT	0.814
	8	MACT	1.106	MACT	0.812	MACT	0.957	MACT	0.887
Starvation-stress	1	RPS11	0.680	TUB	0.247	RPS11	0.282	RPS11/AK	0.372
treatments	2	TUB	0.720	RPS15	0.283	TUB	0.304		
	3	RPS15	0.778	RPS11	0.379	18S	0.480	EF	0.446
	4	18S	0.804	18S	0.506	RPS15	0.506	RPS15	0.521
	5	AK	0.826	AK	0.585	AK	0.624	TUB	0.573
	6	EF	0.896	EF	0.595	EF	0.767	18S	0.645
	7	ACT	0.952	ACT	0.621	ACT	0.785	ACT	0.759
	8	MACT	1.102	MACT	0.736	MACT	1.009	MACT	0.845
All above conditions	1	RPS11	0.946	RPS11	0.463	RPS11	0.370	RPS15/EF	0.488
	2	RPS15	1.011	RPS15	0.504	RPS15	0.655		
	3	TUB	1.037	TUB	0.524	TUB	0.671	TUB	0.611
	4	EF	1.107	EF	0.549	AK	0.806	RPS11	0.666
	5	AK	1.174	AK	0.672	EF	0.832	18S	0.788
	6	18S	1.203	18S	0.694	18S	0.900	AK	0.914
	7	ACT	1.354	ACT	0.842	ACT	1.146	ACT	1.077
	8	MACT	1.372	MACT	0.869	MACT	1.175	MACT	1.151

The expression stability was also measured using the Delta Ct method, BestKeeper, NormFinder, and geNorm and ranked from the most stable to the least stable.

### Ranking of *N. lugens* Reference Genes Over all Treatments

All four programs identified ACT and MACT as the least stable genes, and RPS11 and RPS15 as the most stable genes except geNorm (Table 2). According to RefFinder, the stability ranking from the most stable to the least stable across the different developmental stages, body parts, populations, and stressors was RPS11, RPS15, EF, TUB, AK, 18S, ACT, and MACT (Table S2).

## Discussion

This work analyzed the expression stability of eight candidate reference genes in *N. lugens* across different treatments and developmental stages using qRT-PCR. A major result of this study is that 18S showed unacceptable variation in response to certain treatments. Previously, 18S ribosomal RNA has been considered as an ideal reference gene due to its apparent relatively invariable rRNA expression levels with respect to other genes [38]. 18S rRNA was found to be one of the most suitable housekeepers in the different developmental stages of *Lucilia cuprina* [39], in different organs of *Rhodnius prolixus* under diverse conditions [40], [41], and in the planthopper *Delphacodes kuscheli* infected by the plant fijivirus *Mal de Río Cuarto virus* (MRCV) [42]. However, in our study, 18S ranked as one of the least stable genes in the total samples and almost in all experimental conditions indicating that 18S was not suitable as a reference gene for *N. lugens* under our experimental conditions (Tables S2, S3, S4, S5, S6, S7, S8, S9, S10, S11). This result is in line with the earlier studies indicating that 18S rRNA is not stable enough in *Bactrocera dorsalis* under specified experimental conditions [43]. The transcription by a separate RNA polymerase is proposed to be a reason why rRNA could not be considered as a suitable reference gene [44]. On the other hand, one of the major limitations of using the 18S gene as a normalizer in qRT-PCR is that an imbalance of rRNA and mRNA fractions can occur between samples [38]. Our study suggests that 18S rRNA could not be used for correcting sample-to-sample variation of mRNA quantity in *N. lugens*.

Like 18S rRNA, actin is another commonly used reference gene which encodes a major component of the protein scaffold that supports the cell and determines its shape, and is expressed at moderately abundant levels in most cell types. Actin has been highly ranked as a suitable reference gene in studies of gene expression in *Apis mellifera* [45], *Schistocera gregaria* [46], *Drosophila melanogaster* [47], *Plutella xylostella* [48], and *Chilo suppressalis* [48]. Actin gene has as well been selected as reference gene in gene expression studies in *N. lugens* [12], [13], [14]. However, compared with the other candidate genes examined here, the expression levels of ACT and MACT were highly variable across the different treatments (Tables S2, S3, S4, S5, S6, S7, S8, S9, S10, S11). ACT and MACT, which participate in many important cellular processes including muscle contraction, cell motility, cell division and cytokinesis, ranked one of the least stable genes in the total samples and under almost all experimental conditions. And not surprisingly, its transcript level varies among developmental stages and different cell types, since it has functions in various cellular processes. In *N. lugens*, ACT and MACT should not be used as reference genes under certain treatments.

Our results also demonstrated that the best-suited reference genes can be different in response to diverse factors (Table S2). Reference genes need to be appropriately selected under different experimental conditions. However, the expression of several reference genes from *N. lugens* were comparatively stable across selected experimental conditions. Ranking of the genes differed somewhat for geNorm, NormFinder, BestKeeper, and the delta Ct method probably because the programs have different algorithms and different sensitivities toward co-regulated reference genes. In spite of the slight discrepancies, all the programs identified both RPS11 and RPS15 as the same ideal reference genes for most of the experimental conditions assessed here (Table S2). Ribosomal proteins compose the ribosomal subunits involved in the cellular process of translation in conjunction with rRNA. RPS11 and RPS15 encode the component of the 40S ribosomal subunit which is the small subunit of eukaryotic 80S ribosomes [49]. Considering the function of ribosomal proteins, it is not surprising that their transcription level varies among different cell types and developmental stages in the brown planthopper. Our result is in line with the earlier studies on ribosomal protein genes in *A. mellifera* [45], *S. gregaria* [46], *Tribolium castaneum* [50], [51], *D. melanogaster* [47], *B. mori* [48], *C. suppressalis* [48], and *Bemisia tabaci* [52].

Arginine kinase, which is the only phosphagen kinase in two major invertebrate groups, namely arthropods and mollusks, was one of the most stable genes in *Bombus terrestris* [53]. In our study, AK was also the most stable gene in BPH under extremely low temperature stress (Table S7), and the second most stable gene in nymphs (Table S3). Elongation factor which plays an important role in translation by catalyzing the GTP-dependent binding of aminoacyl-tRNA to the acceptor site of the ribosome exhibited the second most stable expression in the BPH under pesticide-stress (Table S2). EF was found to be the most stable genes for the labial gland and fat body of *Bombus lucorum* [53] and for reliable normalization of qRT-PCR assays studying density-dependent behavioral change in *Chortoicetes terminifera* [54]. However, arginin kinase and elongation factor didn’t show acceptable stable expression in most treatments (Table S2). Even for housekeeping genes, whose products are indispensable for every living cell and are relatively stably expressed, there are tissue-specific differences based upon extra demands in the required rate at which new housekeeping proteins need to be produced to maintain cell function [55].

Multiple reference genes are increasingly used to analyze gene expression under various experimental conditions, because one reference gene is usually insufficient to normalize the expression results of target genes [56]. After measuring the expression of 20 candidate reference genes and 7 target genes in 15 *Drosophila* head cDNA samples using qRT-PCR, 20 reference genes exhibited sample-specific variation in their expression stability and the most stable normalizing factor variation across samples did not exhibit a continuous decrease with pairwise inclusion of more reference genes; these results suggest that either too few or too many reference genes may detriment the robustness of data normalization [57]. When several reference genes are used simultaneously in a given experiment, the probability of biased normalization decreases. GeNorm determines the pairwise variations (V) in normalization factors (the geometric mean of multiple reference genes) using n or n +1 reference genes. Our results showed that the best-suited reference genes were different across different experimental conditions (Figure 2). This implies that the expression stability of putative control genes needs to be verified before each qRT-PCR experiment.

## Conclusion

To our knowledge this is the first study to evaluate candidate reference genes for gene expression analyses in *N. lugens*. Most importantly, we identified reference genes which should be used for accurate elucidation of the expression profiles of functional genes. We concluded that RPS15, RPS11, and TUB were the most suitable reference genes for the analysis of developmental stage, body part, and geographic population, respectively (Table S2). And that RPS15, RPS11, RPS15, and RPS11 were the most suitable reference genes under temperature, pesticide, diet, and starvation stress, respectively (Table S2). This work emphasizes the importance of establishing a standardized reverse-transcription quantitative PCR procedure following the MIQE guidelines in *N. lugens*, and serves as a resource for screening reference genes for expression studies in other insects.

## Supporting Information

Table S1 **Insecticides toxicity to 3^rd^ instar** ***N. lugens*** **larvae.**
(DOC)

Table S2 **Expression stability of the candidate reference genes in the total samples.** The average expression stability of the reference genes was measured using the Geomean method of RefFinder (http://www.leonxie.com/referencegene.php?type=reference). A lower rank indicates more stable expression.
(DOC)

Table S3 **Expression stability of the candidate reference genes across different nymphal stages and across different sexes.** The average expression stability of the reference gene was measured using the Geomean method of RefFinder (http://www.leonxie.com/referencegene.php?type=reference). A lower rank indicates more stable expression.
(DOC)

Table S4 **Expression stability of the candidate reference genes different body parts of female and male adults.** The average expression stability of the reference gene was measured using the Geomean method of RefFinder (http://www.leonxie.com/referencegene.php?type=reference). A lower rank indicates more stable expression.
(DOC)

Table S5 **Expression stability of the candidate reference genes across males and females in the heads, thoraxes, abdomens, and whole bodies.** The average expression stability of the reference gene was measured using the Geomean method of RefFinder (http://www.leonxie.com/referencegene.php?type=reference). A lower rank indicates more stable expression.
(DOC)

Table S6 **Expression stability of the candidate reference genes across two different** ***N. lugens*** **geographic populations.** The average expression stability of the reference gene was measured using the Geomean method of RefFinder (http://www.leonxie.com/referencegene.php?type=reference). A lower rank indicates more stable expression.
(DOC)

Table S7 **Expression stability of the candidate reference genes across different temperatures.** The average expression stability of the reference gene was measured using the Geomean method of RefFinder (http://www.leonxie.com/referencegene.php?type=reference). A lower rank indicates more stable expression.
(DOC)

Table S8 **Expression stability of the candidate reference genes under different pesticide stresses.** The average expression stability of the reference gene was measured using the Geomean method of RefFinder (http://www.leonxie.com/referencegene.php?type=reference). A lower rank indicates more stable expression.
(DOC)

Table S9 **Expression stability of the candidate reference genes of** ***N. lugens*** **fed on different diets.** The average expression stability of the reference gene was measured using the Geomean method of RefFinder (http://www.leonxie.com/referencegene.php?type=reference). A lower rank indicates more stable expression.
(DOC)

Table S10 **Expression stability of the candidate reference genes of** ***N. lugens*** **fed on non-genetically modified rice and genetically modified rice.** The average expression stability of the reference gene was measured using the Geomean method of RefFinder (http://www.leonxie.com/referencegene.php?type=reference). A lower rank indicates more stable expression.
(DOC)

Table S11 **Expression stability of the candidate reference genes of straved** ***N. lugens*** **.** The average expression stability of the reference gene was measured using the Geomean method of RefFinder (http://www.leonxie.com/referencegene.php?type=reference). A lower rank indicates more stable expression.
(DOC)
