# Supplementary material for: Selection and Evaluation of Potential Reference Genes for Gene Expression Analysis in the Brown Planthopper, Nilaparvata lugens (Hemiptera: Delphacidae) Using Reverse-Transcription Quantitative PCR
Source: PLoS One. 2014 Jan 23;9(1):e86503. doi: 10.1371/journal.pone.0086503 (PMC3900570; doi:10.1371/journal.pone.0086503)
Supplement: Table S1 — Insecticides toxicity to 3rd instar N. lugens larvae. (DOC) [file pone.0086503.s001.doc]

**Table S1. Insecticides toxicity to 3^rd^ instar *N. lugens* larvae.**

| **Insecticides** | **N ^a^** | **Slope ± SE ^b^** | **LC_50_ ^c^** | **χ^2^ ^d^** |
| --- | --- | --- | --- | --- |
| nitenpyram | 420 | 1.76± 0.21 | 0.40 (0.28-0.51) | 0.78 |
| pymetrozine | 360 | 2.50± 0.31 | 42.08 (34.03-50.89) | 2.11 |
| buprofezin | 420 | 0.98± 0.17 | 1.19 (0.74-1.74) | 0.49 |
| isoprocarb | 360 | 1.67± 0.25 | 34.91 (27.05-46.33) | 1.10 |
| chlorpyrifos | 360 | 2.48± 0.33 | 52.27 (43.05-66.08) | 2.28 |
| abamectin | 360 | 1.65±0.24 | 2.72 (2.06-3.54) | 0.44 |

**^a^ Number of tested larvae**

**^b^ SE = standard error**

**^c^ LC_50_ = Lethal Concentration 50, the dosage of interest is typically the lethal concentration will kill 50% of the population of organisms in a given period of time. Expressed in mg/L; 95% fiducial limits (FL) of LC_50_ are given in parenthesis, respectively.**

**^d^ Chi-square testing linearity of dose-mortality responses**
