# Supplementary material for: Selection and Evaluation of Potential Reference Genes for Gene Expression Analysis in the Brown Planthopper, Nilaparvata lugens (Hemiptera: Delphacidae) Using Reverse-Transcription Quantitative PCR
Source: PLoS One. 2014 Jan 23;9(1):e86503. doi: 10.1371/journal.pone.0086503 (PMC3900570; doi:10.1371/journal.pone.0086503)
Supplement: Table S2 — Expression stability of the candidate reference genes in the total samples. The average expression stability of the reference genes was measured using the Geomean method of RefFinder (http://www.leonxie.com/referencegene.php?type=reference). A lower rank indicates more stable expression. (DOC) [file pone.0086503.s002.doc]

**Table S2. Expression stability of the candidate reference genes in the total samples.** The average expression stability of the reference genes was measured using the Geomean method of RefFinder (http://www.leonxie.com/referencegene.php?type=reference). A lower rank indicates more stable expression.

| **Rank** | **Developmental stage ^a^** | | **Body part ^b^** | | **Geographic population ^c^** | | **Temperature ^d^** | | **Pesticide ^e^** | | **Diet ^f^** | | **Stravation ^g^** | | **Total ^h^** | |
| --- | --- | --- | --- | --- | --- | --- | --- | --- | --- | --- | --- | --- | --- | --- | --- | --- |
|  | **Genes** | **Geomean of ranking values** | **Genes** | **Geomean of ranking values** | **Genes** | **Geomean of ranking values** | **Genes** | **Geomean of ranking values** | **Genes** | **Geomean of ranking values** | **Genes** | **Geomean of ranking values** | **Genes** | **Geomean of ranking values** | **Genes** | **Geomean of ranking values** |
| 1 | RPS15 | 1.41 | RPS11 | 1.19 | TUB | 1.41 | RPS15 | 1.00 | RPS11 | 1.41 | RPS15 | 1.00 | RPS11 | 1.32 | RPS11 | 1.41 |
| 2 | RPS11 | 2.11 | TUB | 1.86 | RPS11 | 1.68 | TUB | 1.68 | EF | 1.41 | TUB | 2.11 | TUB | 2.11 | RPS15 | 1.68 |
| 3 | TUB | 2.51 | RPS15 | 2.28 | EF | 2.83 | EF | 3.22 | TUB | 2.71 | RPS11 | 2.91 | RPS15 | 3.13 | EF | 2.99 |
| 4 | EF | 3.22 | 18S | 4.23 | RPS15 | 3.41 | RPS11 | 4.36 | RPS15 | 3.72 | EF | 3.66 | AK | 3.34 | TUB | 3.00 |
| 5 | 18S | 4.61 | ACT | 5.48 | AK | 4.40 | AK | 4.95 | 18S | 5.23 | AK | 4.68 | 18S | 4.12 | AK | 4.95 |
| 6 | AK | 5.42 | MACT | 5.57 | ACT | 6.00 | MACT | 5.18 | AK | 5.73 | 18S | 5.73 | EF | 5.05 | 18S | 5.73 |
| 7 | ACT | 7.00 | EF | 7.00 | 18S | 7.48 | 18S | 7.00 | MACT | 7.00 | ACT | 7.00 | ACT | 7.00 | ACT | 7.00 |
| 8 | MACT | 8.00 | AK | 7.44 | MACT | 7.48 | ACT | 8.00 | ACT | 8.00 | MACT | 8.00 | MACT | 8.00 | MACT | 8.00 |

**^a^ Reference gene expression stability at different developmental stages (eggs, 1^st^ instar nymphs, 2^nd^ instar nymphs, 3^rd^ instar nymphs, 4^th^ instar nymphs, 5^th^ instar nymphs, adult females, and adult males)**

**^b^ Reference gene expression stability across different body parts (head, thorax, abdomen, and whole-body of virgin adult males and females)**

**^c^ Reference gene expression stability in two different geographic populations (3^rd^ instar nymphs and adults of Changsha population and Wuhan population)**

**^d^ Reference gene expression stability after treatment of 3^rd^ instar nymphs with different temperatures (4°C, 8°C, 12°C, 16°C, 20°C, 24°C, 28°C, 32°C, 36°C, and 40°C)**

**^e^ Reference gene expression stability after treatment of 3^rd^ instar nymphs with different pesticides (3^rd^ instar nymphs treated with compound pesticide, nitenpyram, pymetrozine, buprofezin, isoprocarb, and chlorpyrifos. A separate control group was used for each pesticide treatment.)**

**^f^ Reference gene expression stability in the *N. lugens* 3^rd^ instar nymphs fed on different diets (artificial diet, TN1, MH63, HH1, SY63, and BTSY63)**

**^g^ Reference gene expression stability in starved *N. lugens* (3^rd^ instar nymphs and adults of starved group and satiation group)**

**^h^ Reference gene expression stability in *N. lugens* under all the specific conditions assessed**
