# Supplementary material for: Selection and Evaluation of Potential Reference Genes for Gene Expression Analysis in the Brown Planthopper, Nilaparvata lugens (Hemiptera: Delphacidae) Using Reverse-Transcription Quantitative PCR
Source: PLoS One. 2014 Jan 23;9(1):e86503. doi: 10.1371/journal.pone.0086503 (PMC3900570; doi:10.1371/journal.pone.0086503)
Supplement: Table S3 — Expression stability of the candidate reference genes across different nymphal stages and across different sexes. The average expression stability of the reference gene was measured using the Geomean method of RefFinder (http://www.leonxie.com/referencegene.php?type=reference). A lower rank indicates more stable expression. (DOC) [file pone.0086503.s003.doc]

**Table S3. Expression stability of the candidate reference genes across different nymphal stages and across different sexes.** The average expression stability of the reference gene was measured using the Geomean method of RefFinder (http://www.leonxie.com/referencegene.php?type=reference). A lower rank indicates more stable expression.

| **Rank** | **Nymph ^a^** | | **Adult ^b^** | |
| --- | --- | --- | --- | --- |
|  | **Genes** | **Geomean of ranking values** | **Genes** | **Geomean of ranking values** |
| 1 | TUB | 1.86 | TUB | 1.57 |
| 2 | AK | 2.00 | RPS15 | 1.86 |
| 3 | RPS15 | 3.08 | RPS11 | 2.59 |
| 4 | RPS11 | 3.31 | AK | 3.66 |
| 5 | ACT | 3.56 | 18S | 4.68 |
| 6 | EF | 5.05 | EF | 5.38 |
| 7 | 18S | 6.44 | MACT | 6.09 |
| 8 | MACT | 7.74 | ACT | 8.00 |

**^a^ Reference gene expression stability in *N. lugens* nymphs was measured by using the raw data of 1^st^, 2^nd^, 3^rd^, 4^th^, and 5^th^ nymphs**

**^b^ Reference gene expression stability in *N. lugens* adults was measured by using the raw data of female adults and male adults**
