# Supplementary material for: Selection and Evaluation of Potential Reference Genes for Gene Expression Analysis in the Brown Planthopper, Nilaparvata lugens (Hemiptera: Delphacidae) Using Reverse-Transcription Quantitative PCR
Source: PLoS One. 2014 Jan 23;9(1):e86503. doi: 10.1371/journal.pone.0086503 (PMC3900570; doi:10.1371/journal.pone.0086503)
Supplement: Table S4 — Expression stability of the candidate reference genes different body parts of female and male adults. The average expression stability of the reference gene was measured using the Geomean method of RefFinder (http://www.leonxie.com/referencegene.php?type=reference). A lower rank indicates more stable expression. (DOC) [file pone.0086503.s004.doc]

**Table S4. Expression stability of the candidate reference genes different body parts of female and male adults.** The average expression stability of the reference gene was measured using the Geomean method of RefFinder (http://www.leonxie.com/referencegene.php?type=reference). A lower rank indicates more stable expression.

| **Rank** | **Female ^a^** | | **Male ^b^** | |
| --- | --- | --- | --- | --- |
|  | **Genes** | **Geomean of ranking values** | **Genes** | **Geomean of ranking values** |
| 1 | RPS11 | 1.00 | RPS11 | 1.73 |
| 2 | TUB | 2.21 | EF | 2.21 |
| 3 | RPS15 | 2.28 | RPS15 | 2.78 |
| 4 | 18S | 4.00 | ACT | 2.83 |
| 5 | MACT | 5.44 | 18S | 3.50 |
| 6 | AK | 6.62 | MACT | 6.24 |
| 7 | ACT | 6.70 | TUB | 6.44 |
| 8 | EF | 6.96 | AK | 8.00 |

**^a^ Reference gene expression stability in *N. lugens* females was measured by using the raw data of head, thorax, abdomen, and whole-body of female adults**

**^b^ Reference gene expression stability in *N. lugens* males was measured by using the raw data of head, thorax, abdomen, and whole-body of male adults**
