# Supplementary material for: Selection and Evaluation of Potential Reference Genes for Gene Expression Analysis in the Brown Planthopper, Nilaparvata lugens (Hemiptera: Delphacidae) Using Reverse-Transcription Quantitative PCR
Source: PLoS One. 2014 Jan 23;9(1):e86503. doi: 10.1371/journal.pone.0086503 (PMC3900570; doi:10.1371/journal.pone.0086503)
Supplement: Table S5 — Expression stability of the candidate reference genes across males and females in the heads, thoraxes, abdomens, and whole bodies. The average expression stability of the reference gene was measured using the Geomean method of RefFinder (http://www.leonxie.com/referencegene.php?type=reference). A lower rank indicates more stable expression. (DOC) [file pone.0086503.s005.doc]

**Table S5. Expression stability of the candidate reference genes across males and females in the heads, thoraxes, abdomens, and whole bodies.** The average expression stability of the reference gene was measured using the Geomean method of RefFinder (http://www.leonxie.com/referencegene.php?type=reference). A lower rank indicates more stable expression.

| **Rank** | **Head ^a^** | | **Thorax ^b^** | | **Abdomen ^c^** | | **Whole-body ^d^** | |
| --- | --- | --- | --- | --- | --- | --- | --- | --- |
|  | **Genes** | **Geomean of ranking values** | **Genes** | **Geomean of ranking values** | **Genes** | **Geomean of ranking values** | **Genes** | **Geomean of ranking values** |
| 1 | TUB | 1.32 | TUB | 1.68 | RPS11 | 1.32 | TUB | 1.57 |
| 2 | EF | 2.78 | RPS15 | 1.86 | EF | 1.68 | RPS15 | 2.28 |
| 3 | AK | 3.22 | ACT | 2.71 | RPS15 | 2.28 | 18S | 2.38 |
| 4 | RPS11 | 3.31 | RPS11 | 3.16 | ACT | 4.43 | RPS11 | 2.38 |
| 5 | RPS15 | 3.50 | AK | 3.94 | 18S | 5.42 | MACT | 5.23 |
| 6 | ACT | 5.09 | MACT | 6.45 | MACT | 5.44 | AK | 6.24 |
| 7 | 18S | 6.73 | EF | 6.88 | TUB | 6.44 | EF | 6.44 |
| 8 | MACT | 7.24 | 18S | 7.24 | AK | 8.00 | ACT | 8.00 |

**^a^ Reference gene expression stability in *N. lugens* heads was measured by using the raw data of females’ and males’ heads**

**^b^ Reference gene expression stability in *N. lugens* thoraxes was measured by using the raw data of females’ and males’ thoraxes**

**^c^ Reference gene expression stability in *N. lugens* abdomens was measured by using the raw data of females’ and males’ abdomens**

**^d^ Reference gene expression stability in *N. lugens* whole-bodies was measured by using the raw data of females’ and males’ whole-bodies**
