# Supplementary material for: Selection and Evaluation of Potential Reference Genes for Gene Expression Analysis in the Brown Planthopper, Nilaparvata lugens (Hemiptera: Delphacidae) Using Reverse-Transcription Quantitative PCR
Source: PLoS One. 2014 Jan 23;9(1):e86503. doi: 10.1371/journal.pone.0086503 (PMC3900570; doi:10.1371/journal.pone.0086503)
Supplement: Table S6 — Expression stability of the candidate reference genes across two different N. lugens geographic populations. The average expression stability of the reference gene was measured using the Geomean method of RefFinder (http://www.leonxie.com/referencegene.php?type=reference). A lower rank indicates more stable expression. (DOC) [file pone.0086503.s006.doc]

**Table S6. Expression stability of the candidate reference genes across two different *N. lugens* geographic populations.** The average expression stability of the reference gene was measured using the Geomean method of RefFinder (http://www.leonxie.com/referencegene.php?type=reference). A lower rank indicates more stable expression.

| **Rank** | **Nymphs of different geographic populations ^a^** | | **Adults of different geographic populations ^b^** | |
| --- | --- | --- | --- | --- |
|  | **Genes** | **Geomean of ranking values** | **Genes** | **Geomean of ranking values** |
| 1 | EF | 1.19 | TUB | 2.11 |
| 2 | RPS11 | 1.41 | AK | 2.99 |
| 3 | TUB | 3.00 | RPS11 | 3.13 |
| 4 | AK | 4.47 | ACT | 3.57 |
| 5 | RPS15 | 4.47 | 18S | 4.33 |
| 6 | MACT | 6.48 | EF | 4.45 |
| 7 | ACT | 6.48 | MACT | 4.76 |
| 8 | 18S | 8.00 | RPS15 | 5.24 |

**^a^ Reference gene expression stability in the nymphs of two different geographic populations was measured by using the raw data of nymphs of Changsha population and Wuhan population**

**^b^ Reference gene expression stability in the adults of different geographic population was measured by using the raw data of adults of Changsha population and Wuhan population**
