# Supplementary material for: Selection and Evaluation of Potential Reference Genes for Gene Expression Analysis in the Brown Planthopper, Nilaparvata lugens (Hemiptera: Delphacidae) Using Reverse-Transcription Quantitative PCR
Source: PLoS One. 2014 Jan 23;9(1):e86503. doi: 10.1371/journal.pone.0086503 (PMC3900570; doi:10.1371/journal.pone.0086503)
Supplement: Table S7 — Expression stability of the candidate reference genes across different temperatures. The average expression stability of the reference gene was measured using the Geomean method of RefFinder (http://www.leonxie.com/referencegene.php?type=reference). A lower rank indicates more stable expression. (DOC) [file pone.0086503.s007.doc]

**Table S7. Expression stability of the candidate reference genes across different temperatures.** The average expression stability of the reference gene was measured using the Geomean method of RefFinder (http://www.leonxie.com/referencegene.php?type=reference). A lower rank indicates more stable expression.

| **Rank** | **Extremely low temperature ^a^** | | **Low temperature ^b^** | | **Proper temperature ^c^** | | **High temperature ^d^** | |
| --- | --- | --- | --- | --- | --- | --- | --- | --- |
|  | **Genes** | **Geomean of ranking values** | **Genes** | **Geomean of ranking values** | **Genes** | **Geomean of ranking values** | **Genes** | **Geomean of ranking values** |
| 1 | AK | 1.32 | RPS11 | 1.57 | TUB | 1.32 | MACT | 2.21 |
| 2 | RPS15 | 2.21 | TUB | 1.73 | RPS15 | 1.97 | TUB | 2.45 |
| 3 | RPS11 | 2.45 | RPS15 | 2.21 | EF | 3.31 | RPS15 | 2.78 |
| 4 | TUB | 3.34 | EF | 3.36 | 18S | 3.72 | AK | 2.78 |
| 5 | EF | 4.23 | ACT | 5.23 | MACT | 4.16 | EF | 3.16 |
| 6 | 18S | 6.00 | 18S | 5.96 | RPS11 | 5.12 | RPS11 | 4.56 |
| 7 | MACT | 7.00 | AK | 6.96 | AK | 6.24 | 18S | 7.00 |
| 8 | ACT | 8.00 | MACT | 7.74 | ACT | 8.00 | ACT | 8.00 |

**^a^ Reference gene expression stability of *N. lugens* in extremely low temperatures was measured by using the raw data of 3^rd^ instar nymphs exposed to 4℃, 8℃, and 12℃ for 5 minutes**

**^b^ Reference gene expression stability of *N. lugens* in low temperatures was measured by using the raw data of 3^rd^ instar nymphs exposed to 16℃ and 20℃ for 5 minutes**

**^c^ Reference gene expression stability of *N. lugens* in average temperatures was measured by using the raw data of 3^rd^ instar nymphs exposed to 24℃ and 28℃ for 5 minutes**

**^d^ Reference gene expression stability of *N. lugens* in high temperatures was measured by using the raw data of 3^rd^ instar nymphs exposed to 32℃, 36℃, and 40℃ for 5 min**
