# Supplementary material for: Selection and Evaluation of Potential Reference Genes for Gene Expression Analysis in the Brown Planthopper, Nilaparvata lugens (Hemiptera: Delphacidae) Using Reverse-Transcription Quantitative PCR
Source: PLoS One. 2014 Jan 23;9(1):e86503. doi: 10.1371/journal.pone.0086503 (PMC3900570; doi:10.1371/journal.pone.0086503)
Supplement: Table S8 — Expression stability of the candidate reference genes under different pesticide stresses. The average expression stability of the reference gene was measured using the Geomean method of RefFinder (http://www.leonxie.com/referencegene.php?type=reference). A lower rank indicates more stable expression. (DOC) [file pone.0086503.s008.doc]

**Table S8. Expression stability of the candidate reference genes under different pesticide stresses.** The average expression stability of the reference gene was measured using the Geomean method of RefFinder (http://www.leonxie.com/referencegene.php?type=reference). A lower rank indicates more stable expression.

| **Rank** | **Compound pesticide ^a^** | | **Nitenpyram ^b^** | | **Pymetrozine ^c^** | | **Buprofezin ^d^** | | **Isoprocarb ^e^** | | **Chlorpyifos ^f^** | |
| --- | --- | --- | --- | --- | --- | --- | --- | --- | --- | --- | --- | --- |
|  | **Genes** | **Geomean of ranking values** | **Genes** | **Geomean of ranking values** | **Genes** | **Geomean of ranking values** | **Genes** | **Geomean of ranking values** | **Genes** | **Geomean of ranking values** | **Genes** | **Geomean of ranking values** |
| 1 | RPS11 | 1.41 | EF | 1.32 | MACT | 1.41 | RPS11 | 1.41 | RPS11 | 1.57 | TUB | 1.41 |
| 2 | TUB | 2.00 | RPS15 | 2.00 | RPS15 | 2.38 | TUB | 1.86 | TUB | 1.86 | RPS15 | 1.73 |
| 3 | MACT | 2.28 | TUB | 2.06 | RPS11 | 2.59 | MACT | 2.91 | EF | 2.63 | EF | 2.21 |
| 4 | EF | 4.14 | RPS11 | 3.72 | TUB | 3.66 | EF | 3.34 | RPS15 | 2.78 | RPS11 | 3.72 |
| 5 | RPS15 | 4.90 | 18S | 5.73 | EF | 3.87 | RPS15 | 3.94 | ACT | 4.73 | AK | 5.00 |
| 6 | ACT | 5.00 | AK | 5.89 | ACT | 4.90 | AK | 6.00 | AK | 6.00 | ACT | 6.00 |
| 7 | AK | 6.48 | ACT | 7.00 | 18S | 7.00 | 18S | 7.00 | 18S | 7.00 | 18S | 7.00 |
| 8 | 18S | 8.00 | MACT | 7.11 | AK | 8.00 | ACT | 8.00 | MACT | 8.00 | MACT | 8.00 |

**^a^ Reference gene expression stability of *N. lugens* treated with compounded pesticide was measured by using the raw data of compound pesticide treatment group and a control group (untreated)**

**^b^ Reference gene expression stability of *N. lugens* treated with nitenpyram was measured by using the raw data of nitenpyram treatment group and a control group (untreated)**

**^c^ Reference gene expression stability of *N. lugens* treated with pymetrozine was measured by using the raw data of pymetrozine treatment group and a control group (untreated)**

**^d^ Reference gene expression stability of *N. lugens* treated with buprofezin was measured by using the raw data of buprofezin treatment group and a control group (untreated)**

**^e^ Reference gene expression stability of *N. lugens* treated with isoprocarb was measured by using the raw data of isoprocarb treatment group and a control group (untreated)**

**^f^ Reference gene expression stability of *N. lugens* treated with chlorpyrifos was measured by using the raw data of chlorpyrifos treatment group and a control group (untreated)**
