# Supplementary material for: Selection and Evaluation of Potential Reference Genes for Gene Expression Analysis in the Brown Planthopper, Nilaparvata lugens (Hemiptera: Delphacidae) Using Reverse-Transcription Quantitative PCR
Source: PLoS One. 2014 Jan 23;9(1):e86503. doi: 10.1371/journal.pone.0086503 (PMC3900570; doi:10.1371/journal.pone.0086503)
Supplement: Table S9 — Expression stability of the candidate reference genes of N. lugens fed on different diets. The average expression stability of the reference gene was measured using the Geomean method of RefFinder (http://www.leonxie.com/referencegene.php?type=reference). A lower rank indicates more stable expression. (DOC) [file pone.0086503.s009.doc]

**Table S9. Expression stability of the candidate reference genes of *N. lugens* fed on different diets.** The average expression stability of the reference gene was measured using the Geomean method of RefFinder (http://www.leonxie.com/referencegene.php?type=reference). A lower rank indicates more stable expression.

| **Rank** | **Artificial diet ^a^** | | **TN1 ^b^** | | **MH63 ^c^** | | **HH1 ^d^** | | **SY63 ^e^** | | **BTSY63 ^f^** | |
| --- | --- | --- | --- | --- | --- | --- | --- | --- | --- | --- | --- | --- |
|  | **Genes** | **Geomean of ranking values** | **Genes** | **Geomean of ranking values** | **Genes** | **Geomean of ranking values** | **Genes** | **Geomean of ranking values** | **Genes** | **Geomean of ranking values** | **Genes** | **Geomean of ranking values** |
| 1 | RPS15 | 1.41 | RPS15 | 1.32 | EF | 1.57 | RPS15 | 1.00 | RPS15 | 1.41 | RPS11 | 2.11 |
| 2 | EF | 2.21 | AK | 2.11 | RPS15 | 2.21 | AK | 2.06 | TUB | 2.63 | 18S | 2.11 |
| 3 | TUB | 2.78 | ACT | 2.83 | TUB | 2.45 | ACT | 3.16 | RPS11 | 3.41 | AK | 3.22 |
| 4 | 18S | 3.46 | EF | 3.22 | RPS11 | 2.51 | EF | 4.00 | EF | 3.66 | TUB | 3.98 |
| 5 | RPS11 | 3.64 | RPS11 | 3.98 | AK | 5.23 | TUB | 4.24 | 18S | 4.23 | EF | 4.43 |
| 6 | AK | 5.96 | TUB | 6.00 | MACT | 6.45 | MACT | 5.92 | AK | 4.28 | ACT | 4.76 |
| 7 | ACT | 6.48 | 18S | 7.24 | 18S | 6.73 | RPS11 | 6.48 | ACT | 5.21 | MACT | 4.79 |
| 8 | MACT | 8.00 | MACT | 7.74 | ACT | 7.00 | 18S | 8.00 | MACT | 7.74 | RPS15 | 5.86 |

**^a^ Reference gene expression stability of *N. lugens* fed on artificial diet was measured by using the raw data of 3^rd^ instar nymphs and adults fed on artificial diet**

**^b^ Reference gene expression stability of *N. lugens* fed on TN1 was measured by using the raw data of 3^rd^ instar nymphs and adults fed on TN1**

**^c^ Reference gene expression stability of *N. lugens* fed on MH63 was measured by using the raw data of 3^rd^ instar nymphs and adults fed on MH63**

**^d^ Reference gene expression stability of *N. lugens* fed on HH1 was measured by using the raw data of 3^rd^ instar nymphs and adults fed on HH1**

**^e^ Reference gene expression stability of *N. lugens* fed on SY63 was measured by using the raw data of 3^rd^ instar nymphs and adults fed on SY63**

**^f^ Reference gene expression stability of *N. lugens* fed on BTSY63 was measured by using the raw data of 3^rd^ instar nymphs and adults fed on BTSY63**
