# Supplementary material for: Selection and Evaluation of Potential Reference Genes for Gene Expression Analysis in the Brown Planthopper, Nilaparvata lugens (Hemiptera: Delphacidae) Using Reverse-Transcription Quantitative PCR
Source: PLoS One. 2014 Jan 23;9(1):e86503. doi: 10.1371/journal.pone.0086503 (PMC3900570; doi:10.1371/journal.pone.0086503)
Supplement: Table S10 — Expression stability of the candidate reference genes of N. lugens fed on non-genetically modified rice and genetically modified rice. The average expression stability of the reference gene was measured using the Geomean method of RefFinder (http://www.leonxie.com/referencegene.php?type=reference). A lower rank indicates more stable expression. (DOC) [file pone.0086503.s010.doc]

**Table S10. Expression stability of the candidate reference genes of *N. lugens* fed on non-genetically modified rice and genetically modified rice.** The average expression stability of the reference gene was measured using the Geomean method of RefFinder (http://www.leonxie.com/referencegene.php?type=reference). A lower rank indicates more stable expression.

| **Rank** | **Non-genetically modified rice^a^** | | **Genetically modified rice^b^** | | **Nymphs on non-genetically modified rice^c^** | | **Adults on non-genetically modified rice^d^** | | **Nymphs on genetically modified rice^e^** | | **Adults on genetically modified rice^f^** | |
| --- | --- | --- | --- | --- | --- | --- | --- | --- | --- | --- | --- | --- |
|  | **Genes** | **Geomean of ranking values** | **Genes** | **Geomean of ranking values** | **Genes** | **Geomean of ranking values** | **Genes** | **Geomean of ranking values** | **Genes** | **Geomean of ranking values** | **Genes** | **Geomean of ranking values** |
| 1 | RPS15 | 1.00 | RPS11 | 1.97 | TUB | 1.32 | RPS15 | 1.19 | RPS15 | 1.68 | 18S | 2.59 |
| 2 | TUB | 1.68 | AK | 2.51 | RPS15 | 1.68 | RPS11 | 1.57 | AK | 1.86 | RPS11 | 2.71 |
| 3 | EF | 3.41 | EF | 3.35 | EF | 2.63 | TUB | 3.31 | TUB | 2.94 | TUB | 2.99 |
| 4 | RPS11 | 3.94 | RPS15 | 3.46 | RPS11 | 3.46 | AK | 3.94 | RPS11 | 3.66 | MACT | 3.72 |
| 5 | AK | 4.47 | MACT | 4.28 | MACT | 5.23 | EF | 5.01 | EF | 3.74 | RPS15 | 3.94 |
| 6 | 18S | 6.24 | TUB | 4.53 | AK | 5.73 | 18S | 5.73 | ACT | 5.18 | EF | 4.14 |
| 7 | ACT | 6.74 | ACT | 4.76 | ACT | 7.24 | ACT | 6.09 | MACT | 6.48 | ACT | 4.76 |
| 8 | MACT | 8.00 | 18S | 6.40 | 18S | 7.74 | MACT | 8.00 | 18S | 8.00 | AK | 5.60 |

**^a^ Reference gene expression stability of *N. lugens* fed on non-genetically modified rice was measured by using the raw data of 3^rd^ instar nymphs and adults fed on TN1, MH63, and SY63**

**^b^ Reference gene expression stability of *N. lugens* fed on genetically modified rice was measured by using the raw data of 3^rd^ instar nymphs and adults fed on HH1 and BTSY63**

**^c^ Reference gene expression stability of nymphs fed on non-genetically modified rice was measured by using the raw data of 3^rd^ instar nymphs fed on TN1, MH63, and SY63**

**^d^ Reference gene expression stability of adults fed on non-genetically modified rice was measured by using the raw data of adults nymphs fed on TN1, MH63, and SY63**

**^e^ Reference gene expression stability of adults fed on genetically modified rice was measured by using the raw data of adults fed on HH1 and BTSY63**

**^f^ Reference gene expression stability of adults fed on genetically modified rice was measured by using the raw data of adults fed on HH1 and BTSY63**
