# Supplementary material for: Selection and Evaluation of Potential Reference Genes for Gene Expression Analysis in the Brown Planthopper, Nilaparvata lugens (Hemiptera: Delphacidae) Using Reverse-Transcription Quantitative PCR
Source: PLoS One. 2014 Jan 23;9(1):e86503. doi: 10.1371/journal.pone.0086503 (PMC3900570; doi:10.1371/journal.pone.0086503)
Supplement: Table S11 — Expression stability of the candidate reference genes of straved N. lugens . The average expression stability of the reference gene was measured using the Geomean method of RefFinder (http://www.leonxie.com/referencegene.php?type=reference). A lower rank indicates more stable expression. (DOC) [file pone.0086503.s011.doc]

**Table S11. Expression stability of the candidate reference genes of straved *N. lugens*.** The average expression stability of the reference gene was measured using the Geomean method of RefFinder (http://www.leonxie.com/referencegene.php?type=reference). A lower rank indicates more stable expression.

| **Rank** | **Nymphs of starvation treatment ^a^** | | **Adults of starvation treatment ^b^** | |
| --- | --- | --- | --- | --- |
|  | **Genes** | **Geomean of ranking values** | **Genes** | **Geomean of ranking values** |
| 1 | RPS11 | 2.00 | RPS11 | 1.00 |
| 2 | EF | 2.34 | TUB | 1.68 |
| 3 | RPS15 | 2.38 | RPS15 | 3.66 |
| 4 | MACT | 3.31 | AK | 3.94 |
| 5 | TUB | 4.05 | 18S | 4.36 |
| 6 | AK | 4.05 | EF | 5.73 |
| 7 | ACT | 7.00 | ACT | 7.00 |
| 8 | 18S | 8.00 | MACT | 8.00 |

**^a^ Reference gene expression stability of starved nymphs was measured by using the raw data of starved nymphs and satiety nymphs fed on SY63**

**^b^ Reference gene expression stability of starved adults was measured by using the raw data of starved adults and satiety adults fed on SY63**
